# Mammographically detected breast clustered microcalcifications localized by chest thin-section computed tomography

**DOI:** 10.1186/s12957-024-03354-0

**Published:** 2024-02-29

**Authors:** Xinjie Liu, Yuhan Bao, Laijian Sui, Jianqiao Cao, Yidan Wang, Chao Yu, Guangdong Qiao, Yizi Cong

**Affiliations:** 1 Surgery Department of West Area, The Affiliated Yantai Yuhuangding Hospital of Qingdao University, 20 Yuhuangding East Road, Yantai, Shandong 264001 P.R. China; 2 Department of Breast Surgery, The Second Hospital of Shandong University, 247 Beiyuan Street, Jinan, Shandong 250031 P.R. China; 3 Department of Orthopedics and Arthrology, The Affiliated Yantai Yuhuangding Hospital of Qingdao University, 20 Yuhuangding East Road, Yantai, Shandong 264001 P.R. China; 4 Department of Breast Surgery, The Affiliated Yantai Yuhuangding Hospital of Qingdao University, 20 Yuhuangding East Road, Yantai, Shandong 264001 P.R. China

**Keywords:** Breast, Multidetector computed tomography, Mammography, Microcalcification

## Abstract

**Background:**

To explore the capability and clinical significance of chest thin-section computed tomography (CT) for localization of mammographically detected clustered microcalcifications.

**Methods:**

A total of 69 patients with 71 mammographically detected clustered microcalcifications received surgical biopsy under the guidance of mammography (MG), CT was used to localize calcifications combined with MG if calcifications can be seen on CT. Intraoperative mammography of the specimens were performed in all cases for identification of the resected microcalcifications. The clinical, imaging and pathological information of these patients were analyzed.

**Results:**

A total of 42 (59.15%) cases of calcifications were localized by CT + MG, 29 (40.85%) cases were guided only by the mammography. All suspicious calcifications on the mammography were successfully removed. Pathological results showed 42 cases were cancer, 23 cases were benign, and 6 cases were atypical hyperplasia. The mean age in the CT + MG group was older than that of the MG group (54.12 vs. 49.27 years; *P* = 0.014). The maximum diameter of clusters of microcalcifications on mammography in the CT + MG group was larger than that of the MG group [(cranio-caudal view, 1.52 vs. 0.61 mm, *P* = 0.000; mediolateral oblique (MLO) view, 1.53 vs. 0.62 mm, *P* = 0.000)]. The gray value ratio (calcified area / paraglandular; MLO, *P* = 0.004) and the gray value difference (calcified area - paraglandular; MLO, *P* = 0.005) in the CT + MG group was higher than that of the MG group. Multivariate analysis showed that the max diameter of clusters of microcalcifications (MLO view) was a significant predictive factor of localization by CT in total patients (*P* = 0.001).

**Conclusions:**

About half of the mammographically detected clustered microcalcifications could be localized by thin-section CT. Maximum diameter of clusters of microcalcifications (MLO view) was a predictor of visibility of calcifications by CT. Chest thin-section CT may be useful for localization of calcifications in some patients, especially for calcifications that are only visible in one view on the mammography.

## Introduction

Breast cancer is the most common malignancy amongst women worldwide [1]. With the increasingly use of breast screening programs, early nonpalpable tumors are becoming more and more commonly detected, and this has resulted in a significant decrease in breast cancer associated mortality in addition to the improvement in therapy. Ductal carcinoma in situ (DCIS), which is typically detected based on the presence of clustered microcalcifications in mammography, is an early stage of breast cancer with an excellent prognosis. Although ultrasound has the capability to visualize large clusters of microcalcifications [2], mammography remains the most important approach for detecting and depicting microcalcifications in the breast, an additionally performed breast MRI could have increased the diagnostic reliability in the assessment of microcalcifications [3].

Clustered microcalcifications may be the only presentation of early breast cancer and usually require the pathological results to guide further treatment [4]. Early-stage breast cancer has attracted notable attention amongst surgeons, and its accurate localization is vital for successful open-surgery or biopsy. When surgical treatment is required, surgeons should perform a tailored resection to accurately remove the lesion whilst aiming for acceptable cosmetic outcomes [5]. For microcalcifications detectable by mammography, the lesions are usually visible from two views on mammography, the cranio-caudal (CC) view and mediolateral oblique (MLO) view. Hook wire-guided localization (WGL), radio‐guided localization (ROLL) or titanium clip with collagen (TCC) can be performed to identify their presence through the guidance of mammography. However, for these procedures, additional manpower and minimal extra X-ray exposures is required for localization of calcifications. A recent study described a noninvasive localization for non-palpable breast microcalcification [6]. They measured the distance between the nipple and the center of the calcification on the CC view and the ML view. The operation proceeded around the intersection between two lines. However, due to the breasts were compressed during mammography examination while were natural status during surgery, so it’s difficult to precise localization of calcification. What’s more, in some rare cases, microcalcifications were too close to the chest wall that standard needle localization could not be seen in the CC projection or other non-standard projection. In this situation, these microcalcifications can’t be localized, increasing the complexity and challenge of biopsy for clinicians. So, for these rare cases where calcifications cannot be localized, it is quite difficult to determine the precise location of calcification and remove the calcifications accurately. Therefore, great efforts are still required to improve the methods currently used for localization of nonpalpable lesions.

Chest CT has become a common imaging modality for evaluating various clinical conditions including diseases of the lung, mediastinum, pleura, chest wall and the diaphragm. Chest CT is of great value in the staging of breast cancer patients with late tumor stage and high recurrence risk factors. Meanwhile, it also contributes to the detection of asymptomatic breast cancer, even when chest CT is performed for detection of other diseases [7–9]. A previous report revealed that preoperative CT could successfully localize breast calcifications [10], indicating its potential value in the localization of breast calcifications.

In order to evaluate the value of chest CT for the localization of microcalcifications, the data of 69 patients with 71 cases of mammographically clustered microcalcifications were collected and analyzed. These patients received preoperative thin-section chest CT scans for tumor staging, regular follow-up of lung lesions or other reasons. Meanwhile, we introduced a 3D method to localize the microcalcifications using CT imaging with the aim of improving of accurate removal of the calcifications.

## Patients and methods

### Patients

The study protocol was approved by the Hospital Human Ethical Committee (2019 − 376). All patients provided signed consent for the study preoperatively. A total of 69 consecutive female patients underwent breast surgical biopsy due to clustered microcalcifications which were detected on a mammography between May 2019 and June 2020. The minimum number of microcalcifications in the cluster was five. Patients who had microcalcification clusters in diffuse or regional distributions were excluded due to difficulties associated with accurate localization of the calcifications.

### Mammography

Digital mammography (Senographe Essential, GE Healthcare, Milwaukee, WI, USA) was performed in all patients. Mammography was obtained in CC and MLO views, and in some special patients, a medio-lateral view. Digital zoom utilising a full-field digital mammography system was used as the performance of digital zoom is comparable to magnification for detecting microcalcifications [11]. Microcalcifications were classified according to the Breast Imaging Reporting and Data System (BI-RADS, 5th edition) on the mammography by one breast radiologist and one breast surgeon, each with > 5 years’ worth of experience of reading mammograms, and all decisions were unanimous. All lesions were categorized as suspected malignancy (BI-RADS 4) or highly suggestive of malignancy (BI-RADS 5).

### CT and calcification localization

**Fig. 1:**
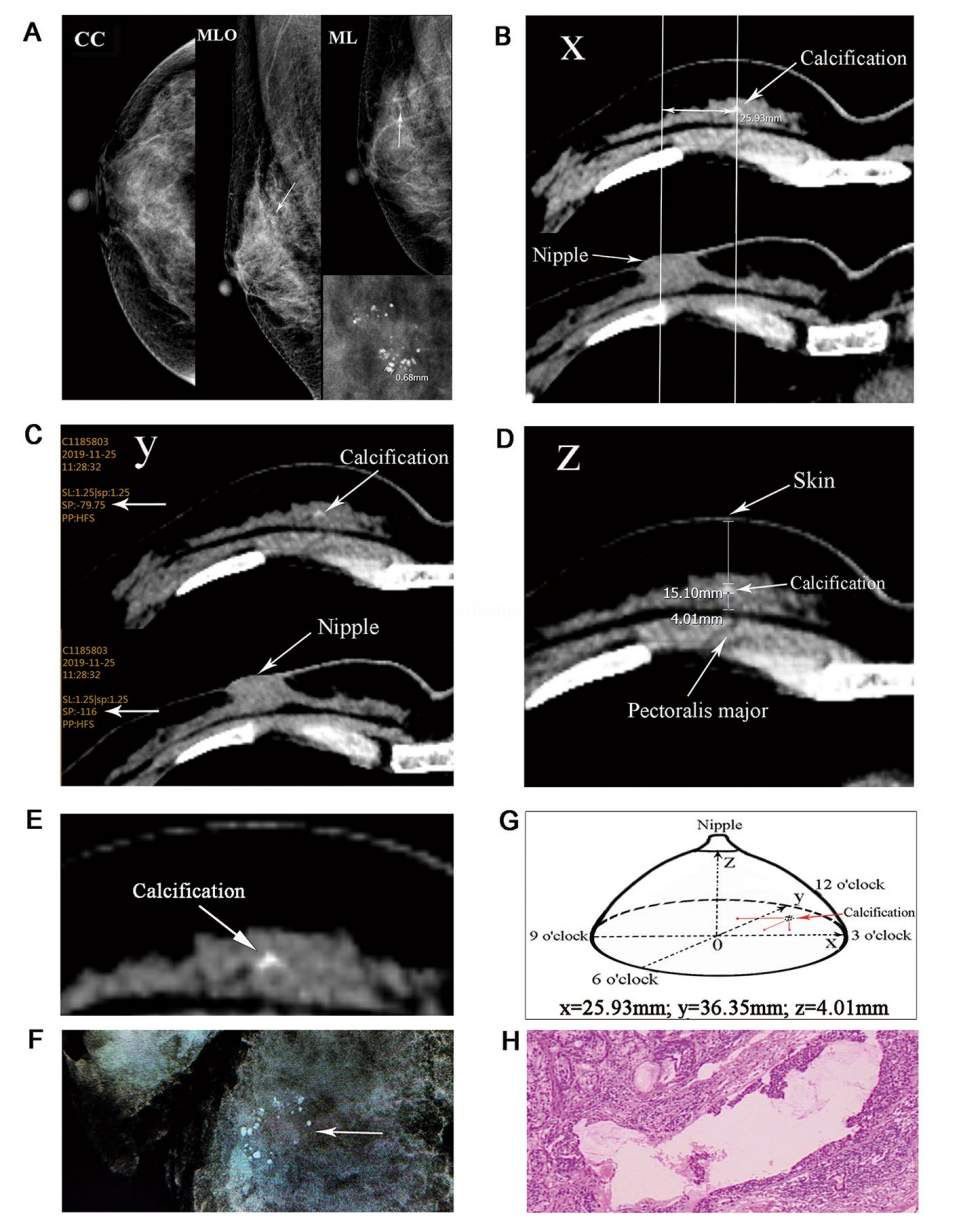
(**A**) Mammographically clustered microcalcifications are visible in the MLO and medio-lateral views in the right breast (arrow) but were not visible in the CC view. The max diameter of calcified nodules was 0.68 mm. (**B**) Transverse chest CT scan adjusted to show the calcifications or the nipple (arrow), the calcifications were measured medial to the nipple (x = 25.93 mm). (**C**) The calcifications were calculated upper to the nipple (y = 36.35 mm). (**D**) The calcifications were measured higher to pectoralis major (z = 4.01 mm) and 15.10 mm below the skin surface. (**E**) Microcalcifications were visible on chest CT scans. (**F**) The calcifications were successfully removed and confirmed by intraoperative mammography. (**G**) 3D diagram of breast calcifications localized by chest CT scans. (**H**) Pathological analysis showing a diagnosis of a low-grade ductal carcinoma in situ (hematoxylin eosin stain, ×40). MLO: mediolateral oblique; CC: cranio-caudal; CT: computed tomography

Chest CT was performed in the supine position, using GE 64-slice CT systems (GE Medical Systems, Milwaukee, WI, USA) with a slice thicknesses of 1.25 mm. The CT protocol is as follows: Scan Type: Pitch&Speed: 0.984:139.37 mm/rot, Rotation time: 0.6 s, Thick:1.25 mm, DFOV:35.0 cm, R/L Center: R1.9 cm, A/P Center: A 0.0 cm, Recon Option: Plus SS50 WW/WL1500/-600 (lung); Plus SS50 WW/WL400/-40 (Mediastinum), Est.max Z location CTD lvol:8.69 mGy, Projected series DLP: 358.79 mGy.cm. The CT results were reported and reviewed by radiologists specialized in reporting on CT examinations. To localize the breast calcifications by CT, the calcifications were initially assessed from the mammography, then they were carefully searched using the mediastinal window of the chest CT. If suspected calcifications were not visible on CT, they were resected only based on the mammography (MG group). If calcifications were visible on CT, then a point was marked on the surface of the breast skin preoperatively following the guidance of CT and mammography (CT + MG group). As shown in Fig. 1, the calcifications were localized in a special patient whose calcifications were visible only in the CC view of the mammogram. The method of localization was as follows: First, the projective point of the middle nipple on the surface of the pectoral muscle was set as the zero position, the direction of the breast 3 o’clock was set as the positive x-axis (Fig. 1B), the direction of breast 12 o’clock was set as the positive y-axis (Fig. 1C), and the zero position to the nipple surface was set as the positive z-axis (Fig. 1D). Slice layers were adjusted and selected on the layer containing the nipple or the calcifications to calculate the length of x, y and z co-ordinates. The calcifications in this case were then measured medial to the nipple (x = 25.93 mm), upper to the nipple (y = 36.35 mm), and higher to the pectoralis major (z = 4.01 mm), 15.10 mm below the skin surface. According to the length of the x and y co-ordinates, a point was marked on the breast skin surface in the same supine position as that used for the CT examination, which indicated the location of the calcifications (Fig. 1G).

### Surgery and pathology

Breast surgical biopsy for suspicious calcifications was performed for all patients. Intraoperative frozen section and hematoxylin and eosin-stained section evaluation was routinely performed for the resected samples. Mastectomy or breast conserving surgery was applied for patients with malignant results. Patients who preserve their breasts must have a negative surgical margin, which means there are no residual in situ or invasive cancers. If the surgical margin is positive, re resection is necessary. For breast cancer patients, immunohistochemical ER, PR, HER2 and Ki67 index were examined. Specimens were HER2 positive when they scored + 3 in the immunohistochemistry or amplification in the fluorescent in situ hybridization analysis.

### Statistical analysis

Statistical analysis was performed using SPSS version 19.0 (IBM Corp). Continuous variables were presented as the mean ± standard deviation. Comparisons between 2 groups were performed using a two-sided t-test when data were normally distributed or a Mann-Whitney U test when not normally distributed. A χ^2^ test and Fisher’s exact test were used to compare categorical variables. Multivariate analyses were performed on variables deemed significant factors (*P* < 0.05) in the univariate analysis. *P* < 0.05 was considered to indicate a statistically significant difference.

## Results

### Characteristics of breast patients with microcalcifications

A total of 69 patients (mean age 52 years, range 36–76 years) with 71 mammographically detected clustered microcalcification underwent surgical biopsy. The calcifications were classified as BI-RADS 4 A (32.39%), BI-RADS 4B (36.62%), BI-RADS 4 C (18.31%) or BI-RADS 5 (12.68%) on the mammography. All suspicious microcalcifications were successfully removed and confirmed by intraoperative mammography. The excised specimen was marked with direction, with a double line marking in the 12 o’clock direction and a single line marking in the 3 o’clock direction. All specimen margins are negative, negative margin is defined as the absence of residual tumor cells. Of these patients, 42 (59.15%) cases of calcifications were localized by CT combined with mammography, and the other 29 (40.85%) cases were guided only by the mammography. The suspected calcifications were more accurately to be removed in the CT + MG group, particularly in 3 of 5 cases where calcifications were only visible in the MLO view (one case shown in Fig. 1). The calcifications were most commonly located in the outer upper quadrant of the breast, accounting for 39.44% (28/71). The average maximum diameter of the microcalcification on MG CC view was 11.22 mm (range 2.41–42.89 mm), and on the MLO view was 11.75 mm (range 2.48–39.51 mm). For calcifications that were visible by CT, the size of calcifications in the CT coronal view was 10.37 mm (range 1.48–32.58 mm), and in the sagittal view was 7.49 mm (range 1.00–41.25 mm). The average maximum diameter of clusters of microcalcifications on the mammography in the CC view was 1.13 ± 1.13 mm, and in the MLO view was 1.15 ± 1.06 mm. Histopathological findings showed there were 42 (59.15%) cases of malignant lesions, 6 (8.45%) cases of atypical hyperplasia, and 23 (32.39%) cases of benign lesions (Table 1).

**Table 1:** Clinicopathological of breast patients with clustered microcalcifications

Characteristics	Total, *n* = 71
Age, years (range)	52.07 ± 8.32 (36–76)
Breast lesions (%)	
Left	39 (54.93%)
Right	32 (45.07%)
Calcification location (%)	
Upper outer quadrant	28 (39.44%)
Upper inner quadrant	12 (16.90%)
Lower inner quadrant	14 (19.72%)
Lower outer quadrant	1 (1.41%)
Inner quadrant	2 (2.82%)
Outer quadrant	2 (2.82%)
Upper quadrant	4 (5.63%)
Central area	6 (8.45%)
Unknown	2 (2.82%)
MG BI-RADS (%)	
4 A	23 (32.39%)
4B	26 (36.62%)
4 C	13 (18.31%)
5	9 (12.68%)
Size of microcalcification area, CC view, mm^ab^ (range)	11.22 ± 7.95 (2.41–42.89)
Size of microcalcification area, MLO view, mm^b^ (range)	11.75 ± 7.79 (2.48–39.51)
Max diameter of microcalcified nodule, CC view, mm^ab^ (range)	1.13 ± 1.13 (0.22–7.55)
Max diameter of microcalcified nodule, MLO view, mm^b^ (range)	1.15 ± 1.06 (0.30–7.36)
Average gray value of calcified area, MLO^b^ (range)	2,552.02 ± 221.32 (1,911.92–3,178.62)
Average gray value of paraglandular, MLO^b^ (range)	2,472.02 ± 218.40 (1,854.66–3,137.37)
Gray ratio, calcified area /paraglandular, MLO^b^ (range)	1.03 ± 0.22 (0.98–1.09)
Gray difference, calcified area - paraglandular, MLO^b^ (range)	80.00 ± 52.95 (-42.05–237.97)
CT localization	
Yes (%)	42 (59.15%)
Size of calcification, coronal view, mm^b^ (range)	10.37 ± 7.89 (1.48–32.58)
Size of calcification, sagittal view, mm^b^ (range)	7.49 ± 8.26 (1.00–41.25)
No (%)	29 (40.85%)
Surgical type (%)	
Wide excision	29 (40.85%)
Breast conserving surgery	6 (8.45%)
Mastectomy ± reconstruction	36 (50.70%)
Tumor size, cm^b^ (range)	1.49 ± 1.09 (0.30–4.50)
Benign	1.14 ± 0.54 (0.50–2.00)
Atypical hyperplasia	0.70 ± 0.54 (0.10–1.50)
Cancer	1.74 ± 1.22 (0.40–4.50)
Pathology (%)	
Benign	23 (32.39%)
Atypical hyperplasia	6 (8.45%)
Cancer	42 (59.15%)

^a^*n*=66. ^b^Mean ± standard deviation. CT: computed tomography; MG: mammography; CC: cranio-caudal; MLO: mediolateral oblique

### Predictive factors for calcifications detected by CT

Although CT is not a standard method for detecting breast calcification, nearly half of the calcifications could be detectable by CT, however, the specific shape of calcified nodules could not be distinguished well on CT, while they mostly presented as a high-density area (Fig. 1E). The mean age of patients in the CT + MG group was higher than that of the MG group (54.12 ± 8.41 years vs. 49.27 ± 7.43 years, *P* = 0.014); there were no significant differences in the other clinicopathological parameters between the two groups (MG-BI-RADS, pathological type, tumor size and surgical type; all *P* > 0.050; Table 2). The maximum diameter of clusters of microcalcifications on the mammography in the CT + MG group was larger than that observed in the MG group (CC view, 1.52 ± 1.34 mm vs. 0.61 ± 0.36 mm, *P* = 0.000; MLO view, 1.53 ± 1.24 mm vs. 0.62 ± 0.34 mm, *P* = 0.000), whereas the area of microcalcification on MG did not differ significantly between these two groups both in the CC and MLO view (all *P* > 0.050). The gray value ratio (calcified area / paraglandular, in the MLO view) in the CT + MG group was higher than that MG group (1.04 ± 0.21 vs. 1.02 ± 0.20, *P* = 0.004). Similarly, the gray value difference (calcified area - paraglandular, MLO) in CT + MG group was also higher compared with the MG group (94.75 ± 51.90 vs. 59.85 ± 48.21, *P* = 0.005) (Table 2).

**Table 2:** Analysis of the different groups of breast patients with microcalcifications

Characteristics	MG group, *n* = 30	CT + MG group, *n* = 41	*P*-value
Age, years^d^	49.27 ± 7.43	54.12 ± 8.41	0.014^a^
Breast lesions (%)			
Left	19 (63.33%)	20 (48.78%)	0.223
Right	11 (36.67%)	21(51.22%)	
MG BI-RADS (%)			
4 A	13 (43.33%)	10 (24.39%)	0.101
4B	12 (40.00%)	14 (34.15%)	
4 C	2 (6.67%)	11(26.83%)	
5	3 (10.00%)	6 (14.63%)	
Size of microcalcification area, CC view, mm^d^	9.12 ± 5.65	12.76 ± 9.05	0.066
Size of microcalcification area, MLO view, mm^d^	11.07 ± 6.56	12.25 ± 8.63	0.533
Max diameter of microcalcified nodule, CC view, mm^d^	0.61 ± 0.36	1.52 ± 1.34	0.000^c^
Max diameter of microcalcified nodule, MLO view, mm^d^	0.62 ± 0.34	1.53 ± 1.24	0.000^c^
Average gray value of calcified area, MLO^d^	2,566.41 ± 229.36	2,541.50 ± 217.51	0.643
Average gray value of paraglandular, MLO^d^	2,506.56 ± 229.95	2,446.75 ± 208.77	0.257
Gray ratio, calcified area /paraglandular, MLO^d^	1.02 ± 0.20	1.04 ± 0.21	0.004^b^
Gray difference, calcified area - paraglandular, MLO^d^	59.85 ± 48.21	94.75 ± 51.90	0.005^b^
Surgical type (%)			
Wide excision	13 (43.33%)	16 (39.02%)	0.350
Breast conserving surgery	4 (13.33%)	2 (4.88%)	
Mastectomy ± reconstruction	13 (43.33%)	23 (56.10%)	
Tumor size, cm^d^	1.12 ± 1.02	1.67 ± 1.11	0.094
Pathology (%)			
Benign	10 (33.33%)	13 (31.71%)	0.898
Atypical hyperplasia	3 (10.00%)	3 (7.32%)	
Cancer	17 (56.67%)	25 (60.98%)	

^a^*P*<0.05, ^b^*P*<0.01, ^c^*P*<0.001. ^d^Mean ± standard deviation. CT: computed tomography; MG: mammography; CC: cranio-caudal; MLO: mediolateral oblique

In the multivariate analysis, the max diameter of clusters of microcalcifications (MLO view, mm) was a significant predictive factor of localization by CT in all patients (*P* = 0.001). The other factors were not considered significant predictive factors (Table 3).

**Table 3:** Multivariate analysis of factors affecting microcalcifications localized by CT in breast patients

Variable	Odds ratio	95% Confidence interval	*P*-value
Age, years	1.084	0.998–1.178	0.056
Max diameter of microcalcified nodule, MLO view, mm	16.886	3.049–93.521	0.001^a^
Max diameter of microcalcified nodule, CC view, mm			0.614
Gray ratio, calcified area /paraglandular, MLO			0.561
Gray difference, calcified area - paraglandular, MLO			0.579

^a^*P*<0.01. CC: cranio-caudal; MLO: mediolateral oblique

### Predictive factors for calcifications detected by CT in breast cancer patients

For breast cancer patients, 59.52% (25/42) cases of calcifications could be detected and localized by CT. 14.29% (6/42) patients received breast conserving surgery, whereas 85.71% (36/42) patients were treated with mastectomy ± reconstruction. Compared with the MG group, the mean age in the CT + MG group was older (*P* = 0.007). The other clinicopathological parameters were not significant predictive factors (pathological type, tumor size, ER, PR, HER2 and surgical type) between the two groups (all *P* > 0.050; Table 4). The average maximum diameter of clusters of microcalcifications on mammography in the CT + MG group was larger than the MG group (CC view, 1.54 ± 1.01 mm vs. 0.70 ± 0.41 mm, *P* = 0.001; MLO view, 1.53 ± 0.95 mm vs. 0.74 ± 0.40 mm, *P* = 0.001). The size of microcalcification area in the MG CC and MLO views also showed difference between these two groups (CC view, *P* = 0.010; MLO view, *P* = 0.039; Table 4).

**Table 4:** Clinicopathological features of breast cancer patients with microcalcifications

Characteristic	MG group, *n* = 17, 40.48%	CT + MG group, *n* = 25, 59.52%	*P*-value
Age, years^c^	50.00 ± 7.82	57.20 ± 8.29	0.007^b^
Size of microcalcification area, CC view, mm^c^	8.97 ± 5.79	16.16 ± 9.67	0.010^a^
Size of microcalcification area, MLO view, mm^c^	10.61 ± 6.16	15.96 ± 8.98	0.039^a^
Max diameter of calcified nodule, CC view, mm^c^	0.70 ± 0.43	1.54 ± 1.01	0.001^b^
Max diameter of calcified nodule, MLO view, mm^c^	0.74 ± 0.40	1.53 ± 0.95	0.001^b^
Average gray value of calcified area, MLO^c^	2,565.16 ± 254.58	2,553.16 ± 168.85	0.855
Average gray value of paraglandular, MLO^c^	2,494.09 ± 254.42	2458.13 ± 175.80	0.590
Gray ratio, calcified area /paraglandular, MLO^c^	1.03 ± 0.22	1.04 ± 0.21	0.140
Gray difference, calcified area - paraglandular, MLO^c^	71.06 ± 54.22	95.03 ± 49.50	0.146
Tumor size, cm^c^	1.23 ± 1.13	2.01 ± 1.20	0.073
Estrogen receptor, %	71.54 ± 32.11	57.59 ± 39.50	0.264
Progesterone receptor, %	54.62 ± 31.52	36.90 ± 32.19	0.126
HER-2 (%)			
Negative	16 (94.12%)	19 (76.00%)	0.122
Positive	1 (5.88%)	6 (24.00%)	
Ki67 (%)	31.54 ± 24.44	29.15 ± 16.21	0.759
Surgical type			
Breast-conserving	4 (23.53%)	2 (8.00%)	0.158
Mastectomy, ± reconstruction	13 (76.47%)	23 (92.00%)	
SLNB (%)			
Negative	13 (76.47%)	19 (76.00%)	0.972
Positive	4 (23.53%)	6 (24.00%)	

^a^*P*<0.05, ^b^*P*<0.01. ^c^Mean ± standard deviation. SLNB: sentinel lymph node biopsy; CT: computed tomography; MG: mammography; CC: cranio-caudal; MLO: mediolateral oblique

In the multivariate analysis, the max diameter of clusters of microcalcifications (MLO view, mm) (*P* = 0.026) and age (*P* = 0.032) were significant predictive factors of localization by CT in breast cancer patients; while none of the other factors were considered predictive (Table 5).

**Table 5:** Multivariate analysis of factors affecting microcalcifications localized by computed tomography in breast cancer patients

Variable	Odds ratio	95% Confidence interval	*P*-value
Age, years	1.125	1.010–1.253	0.032^a^
Max diameter of calcified nodule, MLO view, mm	7.538	1.275–44.583	0.026^a^
Size of microcalcification area, CC view, mm			0.573
Size of microcalcification area, MLO view, mm			0.255
Max diameter of calcified nodule, CC view, mm			0.280

^a^*P*<0.05. CC: cranio-caudal; MLO: mediolateral oblique

## Discussion

Microcalcification is a common feature of both invasive and in situ malignancies of the breast. There are two types of microcalcifications: Type I microcalcifications are composed of calcium oxalate dihydrate and are most frequently present in benign lesions and rarely in breast cancer; whereas type II microcalcifications consisting of calcium phosphates are primarily found in proliferative lesion, such as breast cancer [12]. However, precise differentiation between benign and malignant lesions is difficult due to overlapping mammographic presences of microcalcifications. As an early stage of breast cancer, DCIS usually presents as microcalcifications on mammograms. Accurate lesion localization is mandatory for proper planning of biopsies or breast-conserving surgery with an accurate pathology and a satisfactory cosmetic outcome [13].

In patients with clustered microcalcifications observed in mammograms, image guided histological biopsy should be performed either surgically or percutaneously. Several studies compared different localization techniques for breast tumors that were not palpable. A meta-analysis showed that ROLL and radioactive seed (RSL) were equivalent to WGL in terms of successful excision, although ROLL was associated with improved cosmetic outcomes [14]. ROLL and TCC are equally effective for excision of microcalcifications with clear margins, and exhibited similar re‐intervention rates and resection volumes in breast‐conserving surgery [15]. Reoperation rates and local recurrence-free survival were comparable for ROLL and RSL in patients with breast tumors that were not palpable treated with breast conserving surgery [16]. Among these localization methods, biopsy using WGL based on mammography is commonly performed to examine the microcalcifications. However, WGL have several side effects, including the physical and psychological trauma for patients, reduced accuracy and increased difficulty as patients are required to stand upright and the breast is in a different position compared with surgery [17]. Conversely, for percutaneous breast biopsy, the combination of vacuum-assisted biopsy (VAB) and the standing/upright and prone-type stereotactic mammography systems were relatively highly accurate and decreased the majority of diagnostic surgical biopsies. Jackman et al. [18] reported that failure to retrieve microcalcifications on prone stereotactic breast biopsy was least common on 11-gauge VAB and occurred in only 1% (19/1,423) of lesions. The primary disadvantage of this procedure is the expense of the necessary equipment, the relatively high frequency of vasovagal reactions and the mental discomfort experienced by patients during the biopsy. Nowadays, the majority of institutions have a stereotactic mammography unit in combination with VAB, and this has become a routine biopsy procedure for removal of clustered microcalcifications. However, there are still many hospitals are not equipped with this system owing to the relatively high cost. Combination of wire localization and ultrasound guided VAB is also highly successful (97%) for biopsy of mammographically clustered microcalcifications [19], but special equipment is still required. More important, certain microcalcifications cannot be localized due to them being only visible in only one view of mammography. Therefore, developing a simple and effective method for localization of calcifications is still of great clinical significance.

X-ray is a sensitive and effective method for detecting calcifications. In the present study, we introduced a method using chest thin-section CT to localize the clustered microcalcifications. From our results, 59.15% (42/71) cases of microcalcifications could be localized by chest CT, which may improve the accuracy of resected samples. In breast cancer patients, 59.52% (25/42) cases of calcifications could be localized by CT. In particular, for patients with calcifications only visible on the MG-MLO view, CT provided a relatively accurate location to assist the surgical biopsy. This is a valuable finding which expands the clinical application of chest CT. Previous studies investigated the role of CT, particularly dedicated breast CT, for potential applications in the evaluation of breast lesions. With the wide use of chest CT, serendipitous detection of breast cancer is more frequently being detected, ranging from 0.4 to 2.0% [8, 9]. The malignant features on routine chest CT include rim enhancement of breast tumors, an irregular or spiculated margin or an axillary lymphadenopathy [9, 20]. About 30% of incidental breast lesions were found to be cancers; however, calcification patterns on CT were not diagnostically relevant [20]. The calcifications in the majority of cases only presented as a high-density area on CT in the present study, in agreement with the previous study [20]. A CT device with a higher number of detectors (320) increases the recognition of breast lesions [21], and enhances the value and accuracy in the diagnosis of breast lesions. Enhancement CT reveals additional relevant details to allow for the detection of unsuspected breast lesions [9]. Furthermore, X-ray phase-contrast imaging notably increases soft tissue contrast [22], and has significant potential for improving the diagnosis of breast cancer [23]. Several studies have been performed to improve visualization of microcalcifications using grating-based phase-contrast imaging techniques [24, 25]. Compared to phase-contrast mammography, relatively little information is obtained from phase contrast CT applications for breast imaging. Grating-based phase-contrast computed tomography (GBPC-CT) using a conventional X-ray source in combination with a Talbot-Lau interferometer and an X-ray detector improves depiction quality for the imaging of breast tissue compared to absorption-based imaging and allows for the identification of diagnostically relevant tissue details [26]. Compared with the method based on projection images (AUC = 0.87), based on the distribution of microcalcifications in CT images obtained from the Talbot-Lau interferometer is more effective (AUC = 0.95) for distinguishing benign and malignant breast diseases [27]. For dedicated breast CT, contrast-enhanced cone-beam breast-CT (CE-CBBCT) improved AUC and sensitivity compared with MG and non-contrast-CBBCT (NC-CBBCT), and was even comparable to MRI in dense breast tissue [28]. There was a trend of higher specificity for CE-CBBCT compared with MRI [29]. Another study on CBBCT also demonstrated CBBCT images were comparable to mammograms in calcification identification and may be sufficient for malignant calcifications detection and characterization [30]. For microcalcification (± asymmetry or architectural distortion), there was no difference between the appearance of breast lesions on NC-CBBCT and MG [31]. Together, these data suggest that CT, particularly CBBCT, exhibits promising prospects for the examination of breast lesions.

To the best of our knowledge, there are no large-scale studies evaluating the capability of CT in the localization of breast microcalcifications. In the present study, the average max diameter of clusters of microcalcifications, the gray ratio and gray difference were larger in the CT localization group compared with the control group, suggesting that the larger of the calcified nodules and the higher the density of calcifications, the easier they were to be detected by CT. When calcifications were visible on CT, the size of them on the CT coronal view was very similar with their size on the mammography, but on the sagittal view, the size on CT was smaller than that on the mammography. Dim and fuzzy calcifications usually are not typically visible on chest CT. Multivariate analysis showed that the max diameter of clusters of microcalcifications and age were significant predictive factors of microcalcifications localized by CT in breast cancer patients. These results suggest that the visibility of calcifications on CT is more dependent on the largest particles, and the brightness of calcification may be very important in determining the size of calcifications. The size of calcifications on CT sagittal view is smaller than that in a mammography, which may be due to the fact that the CT scan plane cannot be completely continuous and thus leads to the omission of visible parts of calcifications. Breast density has been notably associated with breast mammographic sensitivity. Dense breast images affect the performance of computer-aided diagnosis [32], suggesting that dense glands also affect the detection of breast microcalcifications. Magnetic resonance imaging can be used to examine dense breasts. In the present study, the age in CT + MG group was older than the MG group, which may be due to younger women having denser breast tissue and block calcifications that are visible by CT.

In the future, with the development of multi-slice CT, GBPC-CT and CBBCT, microcalcifications will be more readily detectable and observed by CT, highlighting the potential clinical value of CT in breast diagnosis. CT localization may be helpful in guiding breast surgery caused by microcalcifications and may also be used to localize titanium clips in breast tissues.

This study was limited by the small size of the patient population. It is necessary to further confirm our findings with a larger number of cases to assess the validity of the results.

## Conclusion

Although CT is currently not used for locating breast lesions, CT exhibits potential in aiding localization of mammographically detected clustered microcalcifications in breast cancer patients. Microcalcifications could be carefully assessed using chest CT for the purpose of localization preoperatively, especially for calcifications that are only visible in one view on the mammography.

## Data Availability

All data relevant for this study are given with the main paper including figures and tables. The raw data that support the findings of this study are available from the corresponding author upon reasonable request.

## Abbreviations

3D: Three dimensional
BI-RADS: Breast Imaging Reporting and Data System
CBBCT: Contrast-enhanced cone-beam breast-CT
CC: Cranio-caudal
CT: Computed tomography
DCIS: Ductal carcinoma in situ
GBPC-CT: Grating-based phase-contrast computed tomography
IDC: Invasive ductal carcinoma
MG: Mammography
MLO: Mediolateral oblique
ROLL: Radio-guided localization
RSL: Radioactive seed
TCC: Titanium clip with collagen
VAB: Vacuum-assisted biopsy
WGL: Hook wire-guided localization
